# Self-Confidence in Women with and without Polycystic Ovary Syndrome

**Published:** 2014-09

**Authors:** Leila Amini, Kobra Valian, Homa Sdeghi Avvalshahr, Ali Montaeri

**Affiliations:** 1 Department of Midwifery, School of Nursing & Midwifery, Iran University of Medical Sciences, Tehran, Iran; 2 Department of Midwifery, School of Nursing & Midwifery, Tehran University of Medical Sciences, Tehran, Iran; 3 Midwifery Department, School of Nursing & Midwifery, Iran University of Medical Sciences, Tehran, Iran; 4 Mental Health Research Group, Health Metrics Research Center, Iranian Institute for Health Sciences Research, ACECR, Tehran, Iran

**Keywords:** Polycystic Ovary Syndrome, PCOS, Self-Confidence, Self-Reliance

## Abstract

**Objective:** To compare self-confidence in woman with and without PCOS according to their ages.

**Materials and methods:** This comparative study was conducted on 400 women (100 with and 300 without PCOS) in clinics of Shahid Akbar-Abadi and Firouzgar Hospitals, from July 2012 to February 2013. SPSS-16 used for statistical analysis (SPSS; SPSS Inc., Chicago, IL, USA).

**Results:** This study showed 98% of PCOS and 93% in non-PCOS groups had average self-confidence with scores of 15-25. None of women in PCOS group and 6.7% of non-PCOS group had high self-confidence (score>25). There was a significant difference between two groups in term of self-confidence level (p< 0.001). There was no significant difference in self-confidence between age group ≤ 30 and age group> 30 in both group, but in PCOS group, self-confidence were significantly higher in both age group under 30 (p<0.0001) and 30 and higher (p<0.0001).

**Conclusion:** Impaired self-confidence in PCOS groups (under 30 and 30 and higher age groups) in comparison with related value of non-PCOS group shows that mental health status in women with PCOS requires urgent psychological attention and support.

## Introduction

Polycystic Ovary Syndrome (PCOS) is one of the common Endocrine disorders of women that affected 5-10 percent of them during the reproductive ages (1). This syndrome has not only long-term effects on health, but also will change the quality of life of these patients (2). The variety of symptoms such as amenorrhea, uterine bleeding, obesity and lack of ovulation is detected. Clinical and metabolic consequences of this syndrome include obesity, insulin resistance and type II diabetes may increase risk of cardiovascular disease, and endometrial or breast cancer. The psychological consequences such as anxiety and depression syndrome can also frequently occur. Other findings are irregular mens, oligo ovulation, hirsutism, acne, and sonographic evidence (1). PCOS also effects on social, lovely and friendly relationships of people (3). Infertility and sterility as one of the negative consequences of PCOS can cause a change in body image. This is due to the loss of sense of control over women's bodies and disrupted body image (4).

In a study in women with PCOS, it has been shown that low self-worth and body image perception in women causes increasing of the anxiety level (5). Physical attractiveness and sexual response changes affect the Self-confidence in these women (6). Self-confidence is the most important determinants of mental health that play an important role in promoting mental health (7).

In other words, cognitive processes, emotion, motivation, decision making and choice, is the result of Self-confidence. Since low Self-confidence has a negative effect on feeling, thought and relationships between people, it requires further attention (8). So this study was done for ascertaining Self-confidence in patients suffered from PCOS in comparison to other women in Tehran, 2012.

## Materials and methods

This comparative study was done in clinics of Shahid Akbar-Abadi and Firouzgar Hospitals, from July 2012 to February 2013. 100 PCOS women and 300 non-PCOS women were selected by consecutive sampling. PCOS diagnosis was based on Rotterdam criteria. Data was collected by demographic questionnaire and Rosenberg Self-confidence questionnaire. This questionnaire has 10 general expressions. Every item has four points from zero to three: I totally disagree(0), I disagree (1), I agree(2), I totally agree (3). Total score will be between 0-25 so that upper than 25 shows high, 15-25 shows moderate, and less than 10 shows low self-confidence (9). This questionnaire was validated by Majdian in Iran (2007) and reported Cronbach’s alpha coefficient 0.93 (10). Also sharifi et al reported Cronbach's alpha coefficient 0.91 (8). Analyzing the data was done by SPSS software 16 (SPSS; SPSS Inc., Chicago, IL, USA).

## Results

In this study the samples were 100 PCOS and 300 non-PCOS women. Mean (SD) of age in PCOS women was 30.28 (6.08) and in non-PCOS women was 29.22 (6.726) years and two groups did not show any statistical significant difference. Mean (SD) of weight in PCOS women was 71.66(8.807) significantly higher than non-PCOS group 60.84(8.73) kilograms (p<0.0001). Also, BMI mean (SD) in affected group was 26.92 (3.648) significantly higher than other group 22.91(3.495) kg/m^2^ (p<0.0001). More information is shown in the table 1. 

Other results of this study showed a significant difference between two groups in term of self-confidence level (P< 0.001). The most women in two groups (98 percent in PCOS and 93 percent in non-PCOS) had average self-confidence with score 15-25. None of women in PCOS group and 6.7 percent of non-PCOS women had high self-confidence (score>25). 

**Table 1:** Demographic characteristics of study groups

**Variables**		**n (%)**		
		**With PCOS** **(n=100)**	**Without PCOS** **(n=300)**	
Age (year)	<30	70 (70)	195(65)	Х^2^=0.839 P=0.360
	≥30	30 (30)	105(35)	
Education(year)	<10	11 (11)	25 (8.3)	Х^2^=2.481 P=0.476
	10-12	51 (51)	175 (58.4)	
	>12	38 (38)	100 (33.3)	
Economic condition	Good	9 (9)	42 (14)	Х^2^=3.177 P=0.204
	Average	72 (72)	241 (80.3)	
	weak	19 (19)	17 (5.7)	
BMI(kg/m^2^)	<20	1 (1)	55 (18.3)	Х^2^=64.54 P=0.0001
	20-25	37 (37)	175 (58.4)	
	25-30	42 (42)	60 (20)	
	≥30	20 (20)	10 (3.3)	
Occupation	Occupied	48 (48)	90 (30)	Х^2^=10.753 P=0.0001
	House wife	52 (52)	210 (70)	

There was no significant difference in self-confidence among under 30 and 30 and higher years women in each group but self-confidence scores in non-PCOS group in comparison with PCOS group were significantly higher in both under 30 (p<0.0001) and 30 and higher (p<0.0001) women (table 2).

**Table 2:** Self-confidence scores according to age in study groups

**Groups** **Age (year)**	**PCOS** **n=100**	**Non-PCOS** **n=300**	
	**Mean± SD**	**Mean± SD**	
<30	20.91 ± 1.68	22.71 ± 1.83	t= 7.205 p<0.0001
≥30	20.46 ± 1.88	22.62 ± 1.78	t=5.758 p<0.0001
	t=1.174 p=0.243	t=0.421 p=0.674	

Table 3 shows more information about each of Rosenberg self-confidence questionnaire.

## Discussion

Results of this study showed most of the women in this study (98percents in PCOS and 93.3 percent in other group) had average Self-confidence. There was a significant difference between two groups in term of self-confidence level (P< 0.001). Self-confidence scores in PCOS group in comparison with non-PCOS group were significantly lower in both under 30 and 30 & higher women. Solati Dehkurdi et al (2006) showed that most of the infertile couple had average Self-confidence (11). The results of McCabe showed that there was a negative correlation between Self-confidence and age in infertile women, according to this study by increasing the age, Self-confidence decreases (12). Also Sharifi Nistank et al in their studies observed a meaningful relation between Self-confidence and age (8).

While the self-confidence is one of the valuable sources and it may repair bodily and spiritual damages (13), in this study we found an important point, none of women with PCOS had high Self-confidence. Since low Self-confidence causes a lot of problems, it is necessary to pay especial attention to this point. 

Nazik et al (2013) in their study found that although PCOS women have a positive body image but their self-esteem is low (14). Bazarganipour et al (2013) in a study on PCOS women found that mediating factors, especially psychologic distress, self-esteem, body image, and sexual function, play an important role in these patients and should be taken into consideration (15). Despite these researches, Morotti et al (2013) believe that some symptoms as moderate hirsutism and hyperandrogenism cannot have any important influence on body image and self-esteem, and sexual function but just in lean PCOS women (16).

In conclusion, since PCOS is a common complex condition associated with psychological, reproductive and metabolic features across the lifespan with a major health burden especially in psychological issues as self-confidence, so we should focus on support, education, and counseling management as well as medical managements in both age groups (less than 30 and 30 and higher). Future research would benefit for reveal influencing factors for any understanding of psychological issues in PCOS women.

**Table 3:** Rosenberg self-confidence questionnaire items in study groups

**Items**	**Absolutely agree**		**Agree**		**Disagree**		**Absolutely disagree**	
	**PCOS** **n=100**	**non-PCOS** **n=300**	**PCOS** **n=100**	**non-PCOS** **n=300**	**PCOS** **n=100**	**non-PCOS** **n=300**	**PCOS** **n=100**	**non-PCOS** **n=300**
	**n(%)**	**n(%)**	**n(%)**	**n(%)**	**n(%)**	**n(%)**	**n(%)**	**n(%)**
Satisfied with myself	10 (10)	62 (20.7)	82 (82)	210 (70)	7 (7)	26 (8.7)	1 (1)	2 (0.7)
I am very good	29 (29)	100 (33.3)	66 (66)	177 (59)	5 (5)	21 (7)	0 (0)	2 (0.7)
Having good as other people.	20 (20)	134 (44.7)	73 (73)	155 (51.7)	6 (6)	8 (2.7)	1 (1)	3 (1)
Able to do as good as other people	29 (29)	117 (39)	66 (66)	167 (55.7)	4 (4)	14 (4.7)	1 (1)	2 (0.7)
Having power & proud of it	4 (4)	73(24.3)	26 (26)	204 (68)	64 (64)	22 (3/7)	6 (6)	1 (0.3)
Being useful	26 (26)	58 (19.3)	57 (57)	176 (7/58)	12 (12)	58 (19.3)	5 (5)	8 (2.7)
Being valuable	15 (15)	105 (35)	76 (76)	180 (60)	9 (9)	13 (3/4)	0 (0)	2 (0.7)
Respect myself	29 (29)	117 (39)	67 (67)	175 (58.3)	4 (4)	8 (2.7)	0 (0)	0 (0)
I am strong	18 (18)	120 (40)	74 (74)	168 (56)	8 (8)	11 (3.7)	0 (0)	0 (0)
Having a positive suggestion to my self	10 (10)	147 (49)	83 (83)	145 (48.3)	5 (5)	8 (2.7)	2 (2)	0 (0)

## References

[1] Banaszewska B, Pawelczyk L, Spaczynski RZ, Duleba AJ (2011). Effects of simvastatin and metformin on polycystic ovary syndrome after six months of treatment. J Clin Endocrinol Metab.

[2] Azziz R, Carmina E, Dewailly D, Diamanti-Kandarakis E, Escobar-Morreale HF, Futterweit W, Et al (2006). Positions statement: criteria for defining polycystic ovary syndrome as a predominantly hyperandrogenic syndrome: an Androgen Excess Society guideline. J Clin Endocrinol Metab.

[3] Laven JS, Imani B, Eijkemans MJ, Fauser BC (2002). New approach to polycystic ovary syndrome and other forms of anovulatory infertility. Obstet Gynecol Surv.

[4] Younesi SJ, Salagegheh A (2001). Body image in fertile and infertile women. J Reprod Infertil.

[5] Benson S, Hahn S, Tan S, Janssen OE, Schedlowski M, Elsenbruch S (2010). Maladaptive coping with illness in women with polycystic ovary syndrome. J Obstet Gynecol Neonatal Nurs.

[6] Russell D, Taylor J (2009). Living alone and depressive symptoms: the influence of gender, physical disability, and social support among Hispanic and non-Hispanic older adults. J Gerontol B Psychol Sci Soc Sci.

[7] Baker L, Gringart E (2009). Body image and self-esteem in older adulthood. Ageing and Society.

[8] Sharifi Neyestanak ND, Ghodoosi Boroojeni M, Seyedfatemi N, Heydari M, Hoseini AF (2012). Self Esteem and its Associated Factors in Patients with Multiple Sclerosis. IJN.

[9] Barkhordary M, Jalalmanesh S, Mahmodi M (2009). The Relationship between Critical Thinking Disposition and Self Esteem in Third and Forth Year Bachelor Nursing Students. Iranian Journal of Medical Education.

[10] Majdian M (2007). Rosenberg self-Esteem scale. AzmonYarPouya.

[11] Solati S, Danesh A, Ganji F, Abedi A (2006). Comparison of self-esteem and coping responses in infertile and fertile couples from Shahrekord, during 2003-2004. J Shahrekord Univ Med Sci.

[12] McCabe MP (2005). Mood and self-esteem of persons with multiple sclerosis following an exacerbation. J Psychosom Res.

[13] Samochowiec J (2010). Self-perception among patients with multiple sclerosis. Arch Psychiatr Psychotherap.

[14] Nazik H, Özdemir F, Nazik E, Arslan S Body image and self-esteem in women with polycystic ovary syndrome.

[15] Bazarganipour F, Ziaei S, Montazeri A, Foroozanfard F, Kazemnejad A, Faghihzadeh S (2013). Predictive factors of health-related quality of life in patients with polycystic ovary syndrome: a structural equation modeling approach. FertilSteril.

[16] Morotti E, Persico N, Battaglia B, Fabbri R, Meriggiola MC, Venturoli S (2013). Body Imaging and Sexual Behavior in Lean Women with Polycystic Ovary Syndrome. J Sex Med.

